# Emotions in self-regulated learning: A critical literature review and meta-analysis

**DOI:** 10.3389/fpsyg.2023.1137010

**Published:** 2023-03-09

**Authors:** Juan Zheng, Susanne Lajoie, Shan Li

**Affiliations:** ^1^Department of Education and Human Services, Lehigh University, Bethlehem, PA, United States; ^2^Department of Educational and Counselling Psychology, McGill University, Montreal, QC, Canada; ^3^Department of Community and Population Health, Lehigh University, Bethlehem, PA, United States

**Keywords:** emotions, self-regulated learning, meta-analysis, review, framework

## Abstract

Emotion has been recognized as an important component in the framework of self-regulated learning (SRL) over the past decade. Researchers explore emotions and SRL at two levels. Emotions are studied as traits or states, whereas SRL is deemed functioning at two levels: Person and Task × Person. However, limited research exists on the complex relationships between emotions and SRL at the two levels. Theoretical inquiries and empirical evidence about the role of emotions in SRL remain somewhat fragmented. This review aims to illustrate the role of both trait and state emotions in SRL at Person and Task × Person levels. Moreover, we conducted a meta-analysis to synthesize 23 empirical studies that were published between 2009 and 2020 to seek evidence about the role of emotions in SRL. An integrated theoretical framework of emotions in SRL is proposed based on the review and the meta-analysis. We propose several research directions that deserve future investigation, including collecting multimodal multichannel data to capture emotions and SRL. This paper lays a solid foundation for developing a comprehensive understanding of the role of emotions in SRL and asking important questions for future investigation.

## 1. Introduction

Students experience a variety of emotions, which can be either beneficial or detrimental to their learning processes and performance. Positive emotions have a considerable impact on students’ academic achievement and can ultimately lead to success in the academic domain (Pekrun et al., 2009). In contrast, negative emotions may impede students’ academic processes. For example, negative emotions (e.g., anger, anxiety, and boredom) have been found to be negatively associated with students’ motivation, learning strategies, and cognitive resources (Pekrun et al., 2002). Given the impressive growth of research on emotions in education, the notion of emotions has been incorporated into various education theories, especially the theoretical frameworks of self-regulated learning (SRL).

Self-regulated learning (SRL) refers to thoughts, feelings, and behaviors that learners plan and adjust to attain learning goals (Zimmerman, 2000). SRL theories account for the cognitive, metacognitive, motivational, and emotional processes and strategies that characterize learners’ efforts to build sophisticated mental models during learning (Pintrich, 2000; Winne and Perry, 2000). Although theorists emphasize different aspects of SRL, the majority of them include emotions as one component of SRL (Boekaerts, 1996; Efklides, 2011). Emotions are generally considered contributing factors that enhance or undermine the use of superficial learning strategies or deep strategies in SRL. A growing number of empirical studies provide general support for the significance of emotions in SRL by examining the effects of emotions on SRL strategies (e.g., Pekrun et al., 2010). However, both theoretical inquiries and empirical evidence about the role of emotions in SRL are still in a state of fragmentation. The field still needs a comprehensive framework that explains the complex connections between emotions and SRL. This paper addresses two research questions*: (1) What theories can be found to explain the complex relationships between academic emotions and SRL? and (2) what empirical evidence exists to support the relationships between academic emotions and SRL*? Our goal is to synthesize the current theoretical frameworks and empirical evidence with the purpose of proposing a model that underpins the link between academic emotions and SRL in individualized learning environments.

## 2. Academic emotions: What are they?

Academic emotion is an important dimension of self-regulated learning that researchers should consider when focusing on within-individual factors influencing learning (Ben-Eliyahu, 2019). Academic emotions are no longer just disruptions that learners should avoid or suppress (Shuman and Scherer, 2014). Academic emotions can be beneficial and harmful, pleasant and unpleasant, and activating and deactivating, depending on the specific emotions and situations.

### 2.1. Taxonomy of emotions

Researchers generally agree to categorize emotions according to the focus of objects (stimulus of emotions), valence (positive or negative), and degree of activation (activating or deactivating). Based on the focus of objects, emotions in the learning context can be distinguished as *achievement emotions, epistemic emotions, topic emotions, and social emotions* (Pekrun and Linnenbrink-Garcia, 2012). This review focuses on individual emotions, i.e., achievement and epistemic emotions that occur in individualized learning environments rather than social emotions that arise in group learning environments.

*Achievement emotions* are emotions that pertain to achievement activities or outcomes that are typically judged by competency-based standards, including anxiety, enjoyment, hope, pride, relief, anger, shame, hopelessness, and boredom (Pekrun, 2006). *Epistemic emotions* are triggered by knowledge and knowledge-generating qualities in cognitive tasks and activities (Trevors et al., 2016; Pekrun et al., 2017). For instance, when personal knowledge conflicts with external knowledge, namely cognitive incongruity, emotions may be activated by the epistemic nature of the task (Muis et al., 2015a). This kind of cognitive incongruity may cause surprise, curiosity, enjoyment, confusion, anxiety, frustration, or boredom. There are overlaps between achievement and epistemic emotions (Pekrun and Stephens, 2012). For example, a student’s enjoyment can be an achievement emotion if it focuses on personal success or an epistemic emotion if it stems from a cognitive incongruity in knowledge. Achievement emotions and epistemic emotions are pervasive in different learning situations and have significant influences on learning (Sinatra et al., 2015). To better understand how these types of emotions can be evaluated in SRL, Rosenberg (1998) suggested that we must also consider the levels and organization of emotions.

### 2.2. Trait emotions and state emotions

According to Rosenberg’s (1998) seminal work, emotions can be distinguished as traits and states. Trait emotions reflect a relatively general and stable way of responding to the world. In contrast, state emotions are characterized as episodic, experiential, and contextual and can be influenced by situational cues (Goetz et al., 2015). These differences can also be applied to the educational context, where one can differentiate trait-like academic emotions from state-like emotions (Pekrun et al., 2002). Trait-like emotions are typical course-related emotional experiences pertaining to a specific course, an exam, or a class. In contrast, state-like emotions are momentary emotional experiences within a single episode of academic life (Ahmed et al., 2013). The differences between trait and state emotions can be traced back to the factors influencing emotions. Trait emotions are derived from memory and are influenced by students’ subjective beliefs and semantic knowledge (Robinson and Clore, 2002). For example, students who do not have abundant knowledge of a specific situation may report more emotions than those with sufficient or similar knowledge. On the other hand, memory plays a less significant role in state emotions (Bieg et al., 2013), where the intra-individual variance of state emotions is influenced more by the students’ interactions between the learning content and environment in a single learning episode. Consequently, these distinctions between trait-like and state-like emotions are essential in understanding the inconsistent self-report emotion measurements that often occur when people are asked to self-report feelings they generally experienced in a course versus those they are currently experiencing (Robinson and Clore, 2002). Furthermore, these distinctions can also help us to better understand the role of emotions in SRL.

## 3. SRL: Two levels of development

Self-regulated learning (SRL) researchers evaluate self-regulated learners based on their theoretical orientations. Winne (1997) first distinguished between an aptitude and an event in terms of the property of SRL. An aptitude is a person’s relatively enduring attribute aggregated in multiple learning activities. For example, a student who reports their habit of memorizing everything in learning can be predicted as a learner who is more inclined to use memorizing strategy. However, this does not mean the student will use a memorizing strategy in every SRL event. An event is a transient and continuous learning state that has a clear starting point and endpoint. Completing a task and finishing an exam are all examples of event-like SRL. Greene and Azevedo (2009) further identified 35 event-like SRL processes at the micro level, e.g., re-reading, reviewing notes, and hypothesizing. Moreover, Efklides (2011) articulated the difference between the Person level SRL, represented by personal characteristics, and the Task × Person level SRL, guided by the monitoring features of task processing. In sum, there exist two levels of SRL: Person level SRL (or aptitude-like SRL) and the Task × Person level SRL (or event-like SRL). The underlying premise for this claim is that different learning contexts, including the nature of tasks and the structure of subjects, can influence how learners regulate their learning process (Poitras and Lajoie, 2013). The claim calls for attention to the acknowledgment of the two levels of SRL while reviewing the theoretical and empirical evidence regarding the role of emotions in SRL.

## 4. What do SRL models say about emotions in SRL?

The answers to the role of emotions in SRL have changed over time because of changing conceptualizations of SRL and the development of SRL models. However, emotions are consistently viewed as an important dimension in SRL (Lajoie, 2008). SRL models paved the way for understanding the role of emotions in SRL. In order to address our first research question regarding emotion and SRL theories, we reviewed five SRL models recognized by Panadero (2017) who argued that all have a consolidated theoretical and empirical foundation. Moreover, the five SRL models are all seminal theories and they are well-recognized in the literature. Järvelä and Hadwin’s (2013) socially shared regulated learning model was not included, as individualized learning is the focus of this paper. Table 1 presents our review of these six SRL models, focusing on what emotions are generated and how emotions affect SRL.

**TABLE 1:** The role of emotions in self-regulated learning (SRL) models.

References	SRL models	Generation of emotions	Effects of emotions
Zimmerman (1990)	Three Cyclical Phases Model	Self-satisfaction in the self-reflection phase	Proactive learners: further efforts to learn; Reactive learners: reduce motivation and efforts to continue
Pintrich (2000)	Four Phases of SRL Model	Anticipatory emotions: test anxiety and fear in the forethought stage	Emotion occurs in the reaction and reflection phase
Boekaerts (1996, 2011)	Adaptive Learning Model	Emotions triggered by appraisal	Influence cognition; Emotion regulation to deal with emotion
Winne and Hadwin (1998)	COPES		The feelings accompanying learning can influence the relations between cognitive conditions and operations
Efklides (2011)	Metacognitive and affective Model of Self-regulated learning (MASRL)	Perceived control and value beliefs influence emotions	Emotions interact with motivation and metacognition in two levels of SRL

As described above, these SRL models reveal a long-established interest in emotion as a key component in SRL. MASRL is the most pertinent theoretical framework that demonstrates an intersection of emotions and SRL with the acknowledgment of the two levels of SRL (i.e., Person and Task × Person level). These models provide some basic assumptions regarding the role of emotions in SRL. In this paper, we critically analyze empirical evidence to find if the literature supports or refutes these theoretical arguments.

Based on the social cognitive paradigm, Zimmerman (1990) acknowledged the existence of emotions and their role in SRL. He described self-satisfaction as a combination of emotions ranging from elation to depression. However, he did not specify which emotions were included in the umbrella of self-satisfaction feelings. Pintrich (2000) extended Zimmerman’s (1990) model and discussed emotions in the context of test anxiety. In Pintrich’s (2000) model, task or contextual features were proposed as factors that might activate test anxiety, and emotion regulation strategies were used to manage test anxiety. This model recognized both the generation and effect of emotions. However, the model only identified test anxiety as an emotion, failing to address other types of emotions that might affect learning. Boekaerts (1996, 2011) gradually shifted her theory from cognition and motivation to emotion and emotion regulation (Panadero, 2017). In her dual-processing model, emotions were proposed as a result of the dual processing of appraisals toward the learning situation (Boekaerts, 2011). If the learning situation were appraised as congruent with personal goals, positive emotions toward the task would be triggered. In contrast, negative emotions would be triggered if the learning situation was appraised as threatening well-being because of task difficulty or insufficient support. The dual processing model highlighted the importance of emotions in SRL but did not specify the type of emotions and the outcomes of emotions in SRL. Winne and Hadwin (1998) emphasized how conditions, operations, products, evaluations, and standards (COPES) could influence the four phases of SRL tasks (i.e., task definition, goals and planning, studying tactics, adaptations). Emotion was not explicitly referred to in this model (Panadero, 2017). The discussion about motivational factors could be an allusion to emotions. Learning feelings may influence the relationship between cognitive conditions and actual operations. In contrast, the metacognitive and affective model of self-regulated learning (MASRL) provided insight into the interactions of metacognition, motivation, and affect in SRL. This model puts more emphasis on the affect in SRL and refers explicitly to the two levels of SRL. As mentioned before, MASRL presented a Person level of SRL functioning, as well as a Task × Person level of SRL events in task processing (Efklides, 2011). We will discuss further how this model describes the relationship between emotions and SRL at two levels.

At the Person level, decisions about what SRL strategies to choose are made based on stable personal characteristics and habitual representation of situational demands (Efklides et al., 2018). Emotion is a relatively stable characteristic of the individual, namely trait emotions. Efklides (2011) describes three extreme scenarios pertaining to how emotions may interact with SRL at the Person level. In the first positive scenario, learners predict success with appropriate SRL strategies and positive emotions. In the second negative scenario, learners predict failure with inappropriate SRL strategies and negative emotions. In the third scenario, learners underestimate or overestimate personal competency; consequently, their emotional reactions and effort expenditure do not match learning outcomes. More specifically, if a student underestimates their mathematics skills, for example, they would emotionally feel anxious and spend more effort learning math, resulting in successful learning outcomes. By contrast, a student who overestimates his effort would experience positive emotions, exert insufficient effort, and have unsuccessful outcomes. These estimated efforts and emotions, restored at a general level, provide cues for subsequent specific tasks (Efklides, 2006).

In a specific task, SRL happens in the form of dynamic events at a Task × Person level. According to this model, task features (e.g., complexity) are objective and independent of a specific learning context but intersect with the person’s attributes and must be considered jointly. The MASRL model proposed three phases of SRL that align with Zimmerman’s (2000) proposition of SRL phases (i.e., *forethought, performance, and self-reflection*). The *forethought phase* may involve two types of cognitive processes. The first type is an automatic and unconscious cognitive process, which can be generated when dealing with familiar, fluent, and effortless tasks (Efklides, 2011). When processes are automatic, emotions are neutral or moderately positive without conscious control processes and increased physiological activity (Carver and Scheier, 1998). The second type of cognitive process is analytic and can be triggered by the task’s structure, novelty, and complexity (Alter et al., 2007). Negative emotions may appear with increased arousal (Efklides, 2011). On the other hand, emotions such as surprise and curiosity may be generated depending on the uncertainty and cognitive interruption that occurs during this phase (Bar-Anan et al., 2009). At the *performance phase*, negative or positive emotions may also change according to the fluency of processing and the rate of progress (Ainley et al., 2005). When tasks are completed, and outcomes are produced at the *self-reflection phase*, positive or negative emotions accompanying the outcomes of the task are triggered or enhanced.

## 5. Academic emotions and SRL: What does the empirical evidence tell us?

To address the second research question (i.e., empirical evidence regarding the role of emotions in SRL), we conducted a comprehensive literature search *via* the PsycINFO, ERIC, and Web of Science databases. The search syntax was (“self-regulated learning” OR “self-regulation” OR “metacognition”) AND (“emotion” OR “affective” OR “anxiety” OR “positive emotions” OR “negative emotions”). The search ended up retrieving 205 articles. We then applied these five inclusion criteria to screen articles: (1) The study must be published in English; (2) The study measured specific self-regulated learning strategies or self-regulated learning processes; (3) The study measured discrete emotions; (4) The study was conducted in a specific learning setting, including an exam, a task, a course, or a specific training program; (5) The study reported the correlation between specific SRL strategies/processes and discrete emotions. Only 23 studies that meet these five criteria are included.

As can be seen in Appendix A, these 23 empirical studies examined the relationship between emotions and SRL strategies. By analyzing the summary of the 23 included studies (Appendix A), we find anxiety, enjoyment, frustration, and boredom are the most frequently examined academic emotions. Metacognitive strategy is most frequently examined in SRL. We then coded these five variables to synthesize the correlation between the four academic emotions and metacognitive strategies. Particularly, there were 14 studies focusing on anxiety, 11 studies on enjoyment, 7 studies on frustration, and 10 studies on boredom. The number of studies was statistically sufficient, based on the rule of a minimum of five independent studies for reliable estimation in the small-sample meta-analysis (Fisher and Tipton, 2015).

**FIGURE 1:**
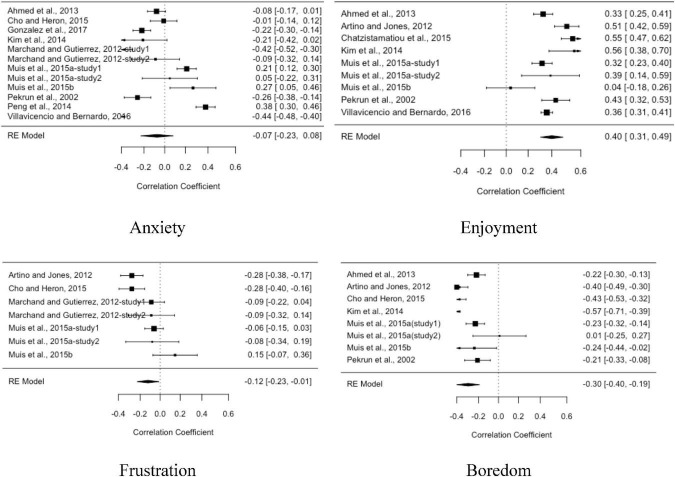
The forest tree of anxiety, enjoyment, frustration, and boredom.

We adopted a random-effects model (Hedges and Vevea, 1998) since the studies in our review differed in methodological characteristics. Among the positive emotions, we found that enjoyment was positively related to metacognitive strategies (*r* = 0.42) (see Table 2). As displayed in the forest tree of enjoyment in Figure 1, we found positive relationships between enjoyment and metacognitive strategies in all studies. In addition to enjoyment, pride also positively predicted cognitive and metacognitive strategies (Ahmed et al., 2013).

**TABLE 2:** Meta-analysis results.

Emotion	*k*	*N*	*r*	95% CI		*r*-range	Test of heterogeneity		
				***LL***	***UL***		***Q***	**df (Q)**	***p***
Anxiety	14	5184	-0.075	-0.23	0.085	−0.44∼0.38	376.76	11	< 0.0001
Enjoyment	9	3362	0.42***	0.32	0.53	0.04∼0.56	42.46	8	< 0.0001
Frustration	7	1396	-0.12*	-0.23	-0.013	−0.062∼-0.28	21.42	6	0.0015
Boredom	10	1902	-0.31***	-0.42	-0.19	−0.57∼0.01	30.37	7	< 0.0001

*N*, sample size; *LL*, lower limits; *UL*, upper limits; *CI*, confidence interval; *p < 0.05, ***p < 0.001.

In terms of negative emotions, anxiety and frustration are generally negatively related to metacognitive strategies (*r* = −0.075 and *r* = −0.12, respectively). However, mixed findings have also been identified across studies. For instance, Muis et al. (2015a) and Peng et al. (2014) found positive relationships between anxiety and metacognitive strategies. Frustration was found to be both positively and negatively related to metacognitive strategies (Artino and Stephens, 2009; Artino and Jones, 2012; Marchand and Gutierrez, 2012; Cho and Heron, 2015). Surprise, curiosity, and confusion are epistemic emotions that produce the most inconsistency in terms of valence categorization, meaning that for learners, they are sometimes pleasant and sometimes unpleasant when experiencing these three epistemic emotions (Noordewier and Breugelmans, 2013). In our meta-analysis, we found boredom negatively related to metacognitive strategies in most of the studies (*r* = −0.31). Curiosity and confusion either positively or negatively predict SRL depending on the depth of strategy use (Muis et al., 2015b). In other words, surprise, curiosity, and confusion can have different effects on shallow processing strategies, deep processing strategies, cognitive strategies, and metacognitive strategies.

The empirical studies provide evidence about the relationship between trait emotions and SRL strategies. Specifically, the majority of the empirical studies focused on examining how academic emotions affect SRL strategies at the Person level (see Appendix A). This emphasis is partly because researchers initially conceptualized SRL as a relatively stable individual inclination, which led to trait-like measures of SRL strategies that have dominated the literature (Boekaerts and Cascallar, 2006). The methodological and ethical issues regarding collecting online data also restrict the exploration of emotions and SRL as states and events (Schutz and Davis, 2000).

To sum up, previous SRL models and empirical studies are not sufficient to reveal the underlying mechanisms of emotions in SRL. One reason is that existing SRL models put unequal emphasis on emotions and SRL. It is worth mentioning that the MASRL model took a crucial step toward a better understanding of emotions in SRL. The model emphasizes both the static and dynamic characteristics of emotions at the two levels of SRL. Nevertheless, the MASRL model provides no clues about how emotions are generated and how the complex interplays of emotions and SRL influence learning outcomes. In terms of empirical evidence, previous studies only addressed the relationships between emotions, SRL strategies, and learning outcomes. Many questions are still unanswered, for instance, (a) what academic emotions will be generated in the SRL process? (b) what are the effects of different emotions in SRL, (c) how do emotions change in different stages of SRL?, and (d) what are the relationships between emotion and SRL at the Task × Person level? An integrated framework is needed to illustrate the role of emotions in SRL better. To substantially advance this field of research, we contend that this framework should address the generation and effects of emotions in SRL. It should provide explanations of the reciprocal relationships between emotions and SRL. Furthermore, the two levels of SRL (i.e., Person and Task × Person level) should be considered to demonstrate how trait and state emotions unfold in different SRL phases, e.g., forethought, performance, and self-reflection.

## 6. Toward an integrated framework for understanding emotions in SRL

**FIGURE 2:**
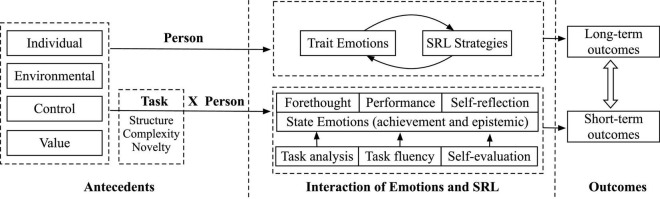
The role of emotions in self-regulated learning (ESRL).

In this study, we proposed an integrative framework of emotions in SRL (ESRL) (Figure 2). The ESRL framework was developed based on the previous conceptualizations of emotions and SRL. It retains the important contributions of previous SRL models. As shown in Figure 2, the framework focuses on the generation and effects of emotions in SRL at two levels (i.e., Person and Task × Person level). In the center of the ESRL framework are the propositions that SRL is an aptitude influenced by trait emotions at the Person level. Moreover, SRL is also an event in a specific task that has dynamic state emotions unfold during different phases at the Task × Person level.

### 6.1. Antecedents of academic emotions

Individual characteristics, environmental factors, control appraisals, and value appraisals are the antecedents of academic emotions at both the Person and Task × Person levels. According to the Control Value theory (CVT), individual antecedents include intraindividual differences such as gender and achievement goals (Pekrun and Perry, 2014). Environmental antecedents (e.g., autonomy support and feedback) are factors that characterize general learning environments. Either trait or state emotions can be triggered depending on the specificity of these antecedents. When control appraisals are conceptualized as the general perception of a learning situation, such as attending online courses (You and Kang, 2014) or a math course (Villavicencio and Bernardo, 2013), control appraisals can predict how students generally feel (trait emotions) in these similar situations. In contrast, when control appraisals are conceptualized as the perception of a specific learning task, for example, solving a math problem (Muis et al., 2015b), control can influence students’ emotions during the problem-solving process (i.e., state emotions). From the perspective of CVT, generalized control-value beliefs can be linked to trait emotions. They can also influence momentary appraisals and state emotions (Pekrun and Perry, 2014).

Furthermore, the majority of the literature in educational psychology has focused on the effect of task features (i.e., task novelty, complexity, and structure) on the occurrence of emotions, especially epistemic emotions (D’Mello and Graesser, 2012; Muis et al., 2018). These task features are objective and independent of a specific learning context but intersect with the person’s attributes and must be considered jointly (Efklides, 2011). Foster and Keane (2015) focused on how new, novel or unique information may trigger surprise if the individual perceives the information as unexpected. D’Mello et al. (2014) proposed that complexity is a crucial antecedent to confusion during learning. Silvia (2010) argued that the complexity of the task would also predict either curiosity or confusion after the surprise toward novelty. In addition to curiosity and confusion, boredom and anxiety are the consequences of task novelty, complexity, and structure. For example, a generally highly competent student may still feel anxious when solving a difficult math question (i.e., task complexity). A student who usually feels bored in a face-to-face math class may be curious about a novel math question that is presented in an innovative way (i.e., task novelty and structure). All the emotions arise from appraisals of uncertainty stemming from task novelty, complexity, or structure (Ellsworth and Scherer, 2003). It is the cognitive disequilibrium underlying uncertainty that plays a critical role in triggering dynamic epistemic emotions (D’Mello and Graesser, 2012). Compared with the dynamic appraisal process of state emotions and ever-changing task attributes, however, trait emotions are relatively stable to interact with SRL.

### 6.2. Interaction between emotions and SRL at the person level

Trait emotions have reciprocal relationships with SRL strategies. Trait emotions are a decontextualized and stable way of reporting feelings (Goetz et al., 2016), while SRL strategies include all the components of SRL, namely cognitive strategies, metacognitive strategies, emotional strategies, and motivational strategies (Warr and Downing, 2000; Ferla et al., 2009). In the interaction between emotions and SRL, trait emotions are presented as an emotional loop or cycle that monitors the strategies or efforts exerted in SRL (Efklides et al., 2018; Ben-Eliyahu, 2019). On the one hand, trait emotions may interfere with students’ prioritization of SRL strategies. Results from empirical studies support these propositions. Positive emotions (e.g., enjoyment, pride) are positively related to students’ usage of cognitive strategies and metacognitive strategies (Pekrun et al., 2002; Artino and Stephens, 2009; Ahmed et al., 2013; Villavicencio and Bernardo, 2013, 2016; Mega et al., 2014; Chatzistamatiou et al., 2015; Chim and Leung, 2016). Villavicencio and Bernardo (2013, 2016) examined the relationship between academic emotions, self-regulation, and achievement in a math course and found that both enjoyment and pride were positively correlated with self-regulation. In terms of negative emotions, boredom, frustration, and anxiety were generally negatively associated with SRL strategies (Pekrun et al., 2002; Kim et al., 2014; Mega et al., 2014; Peng et al., 2014; Gonzalez et al., 2017). More interestingly, researchers found that SRL strategies also influenced students’ trait emotions. For example, Ben-Eliyahu and Linnenbrink-Garcia (2013) examined how self-regulated emotion strategies would influence students’ emotions in academic courses. Results suggested that self-regulated emotions were differentially employed based on course preference, which consequently influences students’ emotions in the course. Furthermore, students who heavily rely on ineffective strategies show prolonged frustration, boredom, and confusion (D’Mello and Graesser, 2012; Sabourin and Lester, 2014; Azevedo et al., 2017). The cyclical effects between emotions and SRL strategies generate long-term effects on student learning outcomes, including persistence (Drake et al., 2014), procrastination (Rakes and Dunn, 2010), and academic achievements (Peng et al., 2014; Gonzalez et al., 2017).

### 6.3. State emotions function at the Task × Person level

Achievement emotions and epistemic emotions are the dominant emotions triggered in a specific task (Pekrun and Stephens, 2012), where these emotions dynamically influence three phases of the SRL cycle (Efklides, 2011). Research provides support for the dynamic emotional changes throughout SRL processes. Anticipatory feelings start from the beginning of a learning activity (i.e., forethought), even though these feelings may be more salient in the self-reflection phase of SRL (Usher and Schunk, 2018). Within the SRL cycle, individuals experience emotions in proportion to the challenges they are facing (Usher and Schunk, 2018). The structure, complexity, and novelty attributes of a task reflect the challenges, which have become the catalyst of academic emotions (Muis et al., 2018). In other words, task analysis in the *forethought phase* predicts the initial emotions students may feel. It is reasonable to anticipate confusion when facing unfamiliar structures, anxiety when facing complexity, and curiosity when facing novelty. In addition to the *forethought phase*, task fluency in the *performance phase* contributes to discrete emotions. In two studies by Winkielman and Cacioppo (2001), students showed more negative emotions in reaction to processing fluency. Fulmer and Tulis (2013) examined students’ fluency and emotions multiple times in a reading task. A latent growth curve showed that positive emotions decline with a decrease in reading fluency. Finally, self-evaluation in the *self-reflection phase* can also affect a change of emotions (Efklides et al., 2018). Learners judge their learning situations by comparing them with performance standards established by themselves and others (Usher and Schunk, 2018). There is no doubt that students experience different emotions even when they have similar performances. A low-performing student may experience more happiness and even pride in the *self-reflection phase* if they consider themself to outperform what they expected. As discussed above, emotions are dynamic and change throughout the three SRL phases, which can influence the effort individuals put toward a task or create an obstacle to further progress in the SRL tasks. Feelings of happiness and pride may lead to renewed efforts, while anxiety and frustration may lead to task avoidance or withdrawal (Usher and Schunk, 2018). Consequently, the short-term learning outcomes will be influenced, including achievements and learning gains, in this SRL event.

### 6.4. Interaction between the two levels of SRL

Self-regulated learning (SRL) is a life-long learning process where students need to plan for each session, each semester, and each training period (Efklides et al., 2018). The short-term learning outcome of one section determines if students will persist with learning or quit on their attempt in the next session. Repeated engagement or disengagement with similar tasks provides consistent information about self-efficacy in a task domain and updates the domain-specific self-concept (Efklides, 2011). Indeed, Metallidou and Efklides (2001) found that self-ratings of confidence and personal estimates of mathematical performance predicted competence at the Person level. It is possible that the short-term perseverance or withdrawal of effort will transfer into long-term outcomes that will be relatively stable over time. From the Task × Person level to Person level, the short-term outcomes of a specific SRL event will gradually influence long-term SRL outcomes. On the other hand, long-term learning outcomes will be transformed into more stable individual characteristics to affect SRL at the Task × Person level. These individual characteristics can be prior knowledge, motivation, and self-efficacy, which will affect how students appraise a specific task. Efklides and Tsiora (2002) conducted a longitudinal study to examine the mechanism between SRL at the Task × Person level and Person level. They found self-concept at the Person level influenced SRL at the Task × Person level. Therefore, from the perspective of life-long learning, we assume the existence of long-term interaction between the two levels of SRL, even though empirical evidence to support this argument is sparse.

## 7. Future directions that build upon the integrative framework

Future research should progress beyond the singular study of relations between emotions and SRL at the Person level. As proposed in our framework, the dynamic relationships between emotion and SRL exist at the Task × Person level. Therefore, it is crucial for future research to examine how emotions unfold in different phases of SRL using advanced methodologies. For example, the high sampling rates of physiological and behavioral measures make it possible to capture the components of SRL with high granularity. Further research in examining emotions and SRL at the Task × Person level could provide insights into the dynamics of SRL, which will consequently inform instruction and the scaffolding for SRL.

Another fruitful area of research resides in the longitudinal research that examines the long-term interplays between the Task × Person and the Person levels of SRL. For example, if students are trained to have proper task analysis skills at the beginning of a specific task, will this kind of training influence their general SRL strategies? Do students transfer the strategies across different contexts and gradually develop them into trait-like personal inclinations? If so, what factors can promote or prohibit this kind of influence? Do trait emotions influence state emotions? Do achievements and motivations associated with a specific task accumulate to influence students’ general persistence and procrastination in learning? Answering these types of questions can provide educators and researchers with tools for designing SRL training programs that can affect learners in the long run.

The third area in need of investigation is how the structure, difficulty, and novelty of a task are related to control-value appraisals and collectively influence academic emotions and SRL. Task difficulty is likely a well-explored direction in task analysis; however, task structure and novelty need further exploration. For example, different emotions and SRL patterns may be triggered by an exam that starts with easy questions or an exam that starts with difficult questions. Thus, a better understanding of the influence of task structures and novelty would contribute to the design of a task that is beneficial for SRL.

Additionally, empirical studies are needed to verify the antecedents of emotions across the three SRL phases. As discussed in our ESRL model, task analysis in the forethought phase, task fluency in the performance phase, and self-evaluation in the self-reflection phase triggers the occurrence and changes of academic emotions. More studies are needed to empirically explore these possibilities. More importantly, researchers can delve into the real-time modeling and visualization of the factors influencing emotions so that corresponding strategies or interventions can be incorporated to optimize the whole learning process. For example, it would be interesting to provide instructors with a dashboard that displays how students’ task fluency evolves and students’ experience of academic emotions over time. In doing so, teachers can provide students with effective emotional or instructional support in real-time.

It is also important to highlight the necessity of using multimodal multichannel data to study SRL for future research. Researchers currently use four types of methodological approaches to studying SRL: (a) self-report measures (i.e., self-report questionnaires, structured dairy, think-aloud/emote aloud, interview); (b) behavioral measures (i.e., facial expressions, body posture, eye-tracking); (c) physiological measures; and (d) computer trace log files. However, each of these four types of measures has its strengths and weaknesses. For example, self-report has its strength in examining SRL at the Person level. However, many self-report measures are static and not capable of capturing the dynamic changes of SRL at the Task × Person level. In contrast, computer log files are powerful in keeping track of SRL at the Task × Person level. However, researchers have to overcome the challenge of making reliable inferences from the trace data.

In response to the drawbacks and strengths of the current methodologies, we argue that researchers need to use multimodal multichannel data when examining the relationship between emotions and SRL at both levels. Self-report measures can be the focus of trait measures at the Person level, whereas physiological measures and trace data can contribute to the situational measures at the Task × Person level. Furthermore, when adopting multichannel data to examine the relationships proposed in the ESRL model, researchers must pay attention to the challenges regarding data analysis and data interpretation. Alignment is the major challenge of analyzing multiple data streams, especially when the starting times are different or the sampling rate varies across devices and methods. For example, it is obvious that physiological sensors need to be attached before an actual data collection process. Consequently, the physiological time stamps start earlier in data collection than in computer log files if both are used for measuring SRL events. In a similar vein, the eye-trackers capture data continuously at 60–120 Hz, but EDA occurs at 8–20 Hz, which means that transformation is necessary when using multimodal multichannel data to analyze different data channels. In terms of data interpretation, it is problematic when researchers cannot make consistent predictions related to specific indices of a method. For example, fixation duration has been interpreted as both cognitive engagement and emotional arousal. These mixed interpretations can cause misleading findings in the literature.

## 8. Conclusion

There is remarkable progress in the theoretical development of SRL toward a holistic understanding of learning or problem-solving process that underscores academic emotions. Although limited empirical studies are available, contemporary literature clearly suggests the complex relationships between academic emotions and SRL. However, this field of study is still scattered and fragmented, given the many ambiguities and arguments about the nature of the two constructs (i.e., academic emotions and SRL). In this study, we contended that emotions could be studied as either traits or states and SRL functions at both Person and Task × Person levels. By reviewing predominant SRL models and analyzing relevant empirical studies, we proposed an integrative framework to explain the role of trait and state emotions in SRL. Specifically, the proposed framework illustrates what the antecedents of emotions are and how they influence academic emotions and consequently SRL and learning outcomes. Moreover, the framework explains how trait emotions influence SRL strategies at the Person level and how state emotions unfold in different phases of SRL at the Task × Person level. We discuss future research directions that build upon our framework, which will advance this field of study considerably. We acknowledge that there is still a long way to go to pinpoint the complex interplays between emotions and SRL. The proposed framework in this study lays a solid foundation for developing a comprehensive understanding of the role of emotions in SRL and asking important questions for future investigation.

## Author contributions

JZ: conceptualization, investigation, and writing–original draft. SLa: writing–reviewing and editing, supervision, and funding acquisition. SLi: writing–reviewing and editing. All authors contributed to the article and approved the submitted version.

## Appendix

**APPENDIX A:** Studies of the relationships between emotions and self-regulated learning (SRL) strategies.

References	Sample	Age	Subjects	Emotions	SRL strategies	Emotion measure	SRL measure	Relationships
Ahmed et al. (2013)	495	12.8	Math	anxiety, boredom, enjoyment, pride	shallow: rehearsal deep: elaboration, organization metacognitive: planning, monitoring, and evaluation	AEQ	MSLQ	Anxiety – Metacognitive; Boredom – Shallow, Metacognitive; Enjoyment ++ Deep, Metacognitive; Pride ++ Shallow, Deep, Metacognitive;
Artino (2009)	481	20.5	Online aviation	boredom, frustration	elaboration metacognitive	AEQ	MSLQ	Boredom – metacognition; Frustration ++ metacognition;
Artino and Jones (2012)	302	20.5	Online aviation	boredom, enjoyment, frustration	elaboration metacognitive	AEQ	MSLQ	Boredom – elaboration, metacognition; Enjoyment ++ elaboration, metacognition; Frustration ++ metacognition;
Ben-Eliyahu and Linnenbrink-Garcia (2013)	250	18.99	General	positive activated, positive deactivated, negative activated, negative deactivated	ERS: reappraisal, suppression, rumination	PANAS	Gross and John (2003)	Reappraisal ++ positive; Reappraisal – negative; Suppression – positive activated; Suppression ++ positive deactivated; Rumination ++ negative; Rumination – positive;
Burić and Sorić (2012)	365	16	Test	hope, hopeless	Volitional strategies: self-efficacy enhancement, negative-based incentives,	AEQ	AVSI	self-efficacy enhancement – hopeless; self-efficacy enhancement ++ hope; negative-based incentives – hope; negative-based incentives ++ hopeless;
Chatzistamatiou et al. (2015)	344	11–12	Math	enjoyment	cognitive: memorization, deep comprehension metacognitive: metacognition, reflection	Price and Mueller (1981)	Dermitzaki and Efklides (2003)	enjoyment ++ cognitive, metacognitive;
Chim and Leung (2016)	180	NA	English language	boredom anxiety enjoyment, anger, Shame, Pride, hopelessness	memory strategy, goal setting, self-evaluation, seeking assistance, environment structuring, learning responsibility, organization	AEQ	Magno (2010)	enjoyment, pride, hope ++ All except environment structuring; anxiety, anger, shame hopelessness ++ All except environment structuring; boredom – all except environment structuring;
Cho and Heron (2015)	229	21.64	Online math	test anxiety, boredom, frustration	metacognitive, CT	AEQ, MSLQ	MSLQ	Boredom – metacognitive, CT; Frustration – metacognitive, CT;
Gonzalez et al. (2017)	520	16.81	Physics	hope, anxiety	metacognitive: planning, monitoring, evaluation	AEQ	Physics Metacognitive Inventory	hope ++ planning, monitoring, evaluation; anxiety – planning, monitoring, evaluation;
Kim et al. (2014)	72	16.7	Online math	boredom, anxiety enjoyment, anger, shame, pride, hopelessness	cognitive strategy self-regulation	AEQ	MSLQ	boredom, anger, shame, hopelessness – self-regulation; enjoyment, pride – self-regulation, cognitive strategy; boredom – cognitive strategy;
Van Nguyen et al. (2015)	623	20.92	Medical students	depression, anxiety, stress	rehearsal, elaboration, CT, organization, metacognitive, time and study environment, help seeking, peer learning, and effort regulation	DASS	MSLQ	help-seeking – depression; time and study environment ++ depression;
Kesici et al. (2011)	320	21.28	Statistics	anxiety	rehearsal, elaboration, CT, organization, metacognitive, time and study environment, help seeking, peer learning, and effort regulation	STARS	MSLQ	effort regulation, help seeking strategies ++ anxiety;
Marchand and Gutierrez (2012)	291	33.5	Research methods course	hope frustration, anxiety	learning strategies	AEQ	(Greene et al., 2004)	hope, anxiety ++ learning strategies; frustration – learning strategies;
Mega et al. (2014)	5805	22.46	General	positive emotions, negative emotions	SRL strategies: organization, elaboration, self-evaluation, metacognition	emotion questionnaire	MSLQ	positive emotions ++ SRL strategies; negative emotions – SRL strategies;
Muis et al. (2015a)	439	21.77	climate change	surprise, curiosity, enjoyment, confusion, anxiety, frustration, boredom	metacognitive, elaboration, CT, rehearsal	EEQ	MSLQ	Surprise – CT; Boredom – rehearsal; curiosity, confusion, enjoyment ++ metacognitive; curiosity, anxiety ++ CT; curiosity, enjoyment ++ elaboration; enjoyment ++ rehearsal;
Muis et al. (2015b)	79	11	Math	surprise, curiosity, enjoyment, confusion, anxiety, frustration, boredom	planning and goal setting, shallow strategies deep strategies metacognitive strategies	EEQ	think-aloud	curiosity, anxiety, frustration ++ shallow; surprise, confusion – shallow; surprise, confusion, boredom – deep; surprise, boredom – planning and goal setting; curiosity, anxiety ++ metacognitive; boredom – metacognitive;
Pekrun et al. (2002)	230	NA	General	enjoyment, hope, anger, anxiety, boredom	elaboration, rehearsal, self-regulation	AEQ	MSLQ	enjoyment, hope ++ elaboration; anger, anxiety, boredom – rehearsal; enjoyment, hope ++ self-regulation; anxiety, boredom – self-regulation;
Peng et al. (2014)	438	15–16	Test	anxiety	metacognitive strategies	TTSQ	TTSQ	anxiety ++ metacognitive;
Villavicencio and Bernardo (2013, 2016)	1345	16.49	Math	anxiety, enjoyment, pride	self-regulation	AEQ	MSLQ	enjoyment, pride ++ self-regulation
Lajoie et al. (2018)	43	24.5	Clinical reasoning	positive activating, negative activating, positive deactivating, negative deactivating)	SRL processes (forethought, performance, self-reflection)	Medical emotions	Log file	Forethought – negative activating emotions Performance – negative deactivating emotions Self-reflection positive activating emotions
Rienties et al. (2019)	1035	First-year	Introductory math, statistic	Epistemic emotions, achievement emotions	SRL strategies	EES, AEQ	Vermunt’s student learning pattern (ILS)	Emotions differ among SRL groups
Taub et al. (2021)	65	21.8	Biology (circulatory system in Metatutor)	surprise, contempt, confusion, frustration	Metacognitive judgments	Facial expressions	Self-report	Surprise – metacognitive accuracy Frustartion ++ Cognitive accuracy.

++ refers to positive relations and – refers to negative relations.
